# Effects of Dementia Care Mapping on well‐being and quality of life of older people with intellectual disability: A quasi‐experimental study

**DOI:** 10.1111/jar.12576

**Published:** 2019-03-13

**Authors:** Feija D. Schaap, Geke J. Dijkstra, Roy E. Stewart, Evelyn J. Finnema, Sijmen A. Reijneveld

**Affiliations:** ^1^ Research Group Living, Wellbeing and Care for Older People NHL University of Applied Sciences Leeuwarden The Netherlands; ^2^ Department of Health Sciences, Community & Occupational Medicine University Medical Center Groningen, University of Groningen Groningen The Netherlands; ^3^ Department of Health Sciences, Applied Health Research University Medical Center Groningen, University of Groningen Groningen The Netherlands

**Keywords:** dementia, DCM, effect, intellectual disability, person‐centred care, quality of life

## Abstract

**Background:**

The ageing of people with intellectual disability, accompanied with consequences like dementia, challenges intellectual disability‐care staff and creates a need for supporting methods, with Dementia Care Mapping (DCM) as a promising possibility. This study examined the effect of DCM on the quality of life of older people with intellectual disability.

**Methods:**

We performed a quasi‐experimental study in 23 group homes for older people with intellectual disability in the Netherlands, comparing DCM (*n* = 113) with care‐as‐usual (CAU; *n* = 111). Using three measures, we assessed the staff‐reported quality of life of older people with intellectual disability.

**Results:**

DCM achieved no significantly better or worse quality of life than CAU. Effect sizes varied from 0.01 to −0.22. Adjustments for covariates and restriction of analyses to people with dementia yielded similar results.

**Conclusion:**

The finding that DCM does not increase quality of life of older people with intellectual disability contradicts previous findings and deserves further study.

## 1 BACKGROUND

In the past few decades, the lifespan of people with intellectual disability has greatly increased. In this population, age‐related conditions like dementia are experienced earlier and are more prevalent than in the general population (Haveman et al., 2010; Heller & Sorensen, 2013). Moreover, pre‐existing deficits and different presentation in adults with intellectual disability make diagnosis of dementia complex. Among people with intellectual disability, its prevalence is estimated to be 18% at the age of 65 (Strydom, Livingston, King, & Hassiotis, 2007). This prevalence is even higher among people with Down's syndrome, 68%–80% of whom have developed dementia by the age of 65 (Coppus et al., 2008; Dekker et al., 2015). In fact, in this group, the average age of onset of dementia is in the early 50s, much sooner than in the general population (Strydom, Chan, King, Hassiotis, & Livingston, 2013; Strydom et al., 2010).

Also in people with intellectual disability, dementia leads to a wide range of changes in memory, functional capacity, communication, neurology, personality and behaviour (Cleary & Doody, 2017). These changes can result in behaviour like agitation, resistance, depression and apathy; responses which present a challenge to care staff (Ball, Holand, Treppner, Watson, & Huppert, 2008; Duggan, Lewis, & Morgan, 1996; Emerson, 2001; Sheehan, Ali, & Hassiotis, 2014). Hence, ageing, and especially dementia, strongly impacts the lives of people with intellectual disability, as well as their housemates and care staff (Janicki & Keller, 2012; Patja, Iivanainen, Vesala, Oksanen, & Ruoppila, 2000; Shooshtari, Martens, Burchill, Dik, & Naghipur, 2011).

Although care staff are a key source of support for older people with intellectual disability (Carling‐Jenkins, Torr, Iacono, & Bigby, 2012; Dodd, 2014), they often feel they lack skills to deal with the increasing complexity of care for their clients (Cleary & Doodey, 2016; Iacono, Bigby, Carling‐Jenkins, & Torr, 2014; Janicki, 2011; Watchman, 2008; Wilkinson et al., 2005). Knowledge and skills from regular geriatric‐ and dementia care could be useful in care for older people with intellectual disability in general, and even more in cases of dementia (Bickenbach et al., 2012; Campens et al., 2017; Hales, Ross, & Ryan, 2006; Iacono et al., 2014; McCarron et al., 2010). Traditionally, care for people with intellectual disability has focused on promoting their well‐being, learning and development of skills (Balogh et al., 2016; Bertelli, Salerno, Rondini, & Salvador‐Carulla, 2017; Leutz, 1999). The ageing of the people with intellectual disability (and dementia) has led to a need for more care and for a more integrated and person‐centred approach, which can be derived partly from standard geriatric and dementia care (Bickenbach et al., 2012; Campens et al., 2017; Hales et al., 2006).

Tom Kitwood introduced the philosophy of personhood in dementia care to change its focus to a person‐centred approach (Barker & Board, 2012; Brooker & Latham, 2015). Evidence suggests that person‐centred methods increase the quality of intellectual disability care and are associated with psychosocial benefits and greater well‐being among older people with intellectual disability (Bertelli et al., 2017; Brown et al., 2016; Brownie & Nancarrow, 2013; Cleary & Doody, 2017; Van der Meer, Nieboer, Finkenflügel, & Cramm, 2018; De Vreese et al., 2012).

**Figure 1:**
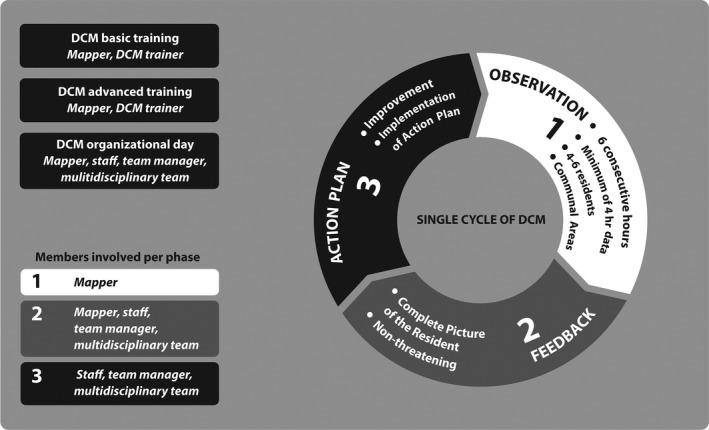
Dementia Care Mapping intervention components and cycle (based on: Van de Ven (2014)

One such person‐centred method is DCM. DCM was designed to support dementia‐care staff working in psychogeriatric nursing homes to improve the quality and effectiveness of care from a person‐centred approach, and thereby improving the well‐being and quality of life of clients with dementia (see Box 1, Figure 1) (Kitwood, 1992). Studies on DCM applied in nursing home settings found less affective behaviour, and physical and verbal agitation in people with dementia (Kuiper, Dijkstra, Tuinstra, & Groothoff, 2009; Rokstad et al., 2013). The method was shown to be applicable, as well as a useful and valuable support to staff caring for people with intellectual disability, whether or not they had dementia (Finnamore & Lord, 2007; Jaycock, Persaud, & Johnson, 2006; Persaud & Jaycock, 2001). Schaap, Fokkens, Dijkstra, Reijneveld, and Finnema (2018) concluded that for older people with intellectual disability, both with and without dementia, DCM was feasible when tailored to daily intellectual disability‐care practices regarding the case histories and examples (Schaap, Fokkens et al., 2018).

Box 1 **Structure and contents of DCM**

Dementia Care Mapping (DCM) is an intervention developed by the Dementia Research Group at Bradford University (UK) to improve the quality and effectiveness of care from the perspective of people with dementia (Brooker & Surr, 2005). It is based on Kitwood's social–psychological theory of personhood in dementia (Kitwood, 1992). DCM was designed as an observational tool to develop person‐centred care for people with dementia in nursing homes (Van de Ven et al., 2013). Person‐centred dementia care can be specified as: valuing people with dementia; using an individual approach that recognizes the uniqueness of the person; making an effort to understand the world from the perspective of the person; and providing a supportive social environment (Brooker, Woolley, & Lee, 2007). DCM has three main components:

**Mappers’ training in DCM**

A staff member receives training to become a certified DCM mapper. A basic DCM mapper's course includes four days of basic concepts and skills. To participate in research, a mapper must achieve the level of advanced mapper. Requirement is a three‐day course focused on the background and theory of DCM and person‐centred care. An advanced DCM mapper can observe(map) care with an inter‐reliability score of ≥0.8, report the observation, provide feedback and instruct staff in drawing up action plans (Van de Ven et al., 2013).

**Organizational introductory briefing**

Before the mapping (systematic observation of the actual care) takes place, the staff of a group home receives a short introduction (two hours). This introduction explains the basic principles of DCM and person‐centred care to ensure endorsement and appropriate implementation (Van de Ven et al., 2013).

**DCM cycle: observations feedback action plan**

The introductory DCM organizational briefing day is followed by a DCM cycle, consisting of:

1. ***Observation, analysis and report.*** A mapper observes four to six residents in communal areas for 4 to 6 consecutive hours. Each 5‐min time frame, a code is noted to record what happened to each resident and the associated behaviour of the staff. The DCM coding protocol contains 23 behavioural category codes (BCCs), well‐/ill‐being (WIB) values, personal detractions (PDs) and personal enhancers (PEs) (Brooker & Surr, 2005).
2. ***Feedback*.** The results of the mapping are communicated to the staff. The purpose of this feedback is to observe residents’ behaviour in the context of both their lives and the care (Brooker & Surr, 2005). Feedback is presented in a non‐threatening way and intended to raise staff awareness of their own and residents’ behaviour, thereby motivating them to improve their competences and performance (Van de Ven et al., 2013).
3. ***Action plans.*** Based on the feedback, the staff draws up action plans to improve care at individual and group levels. Action plans are tools to implement in daily practice the principles of person‐centred care.

Nevertheless, although DCM is feasible and is perceived as valuable in intellectual disability care, evidence on its effectiveness is still lacking (Schaap, Dijkstra, Finnema, & Reijneveld, 2018; Schaap, Fokkens et al., 2018). The aim of this study was therefore to examine the effect of DCM, compared to care‐as‐usual, on the well‐being and quality of life of older clients with intellectual disability.

## 2 METHODS

### 2.1 Study design

To assess well‐being and quality of life in older people with intellectual disability, we performed a quasi‐experimental study from November 2014 to April 2016, comparing DCM with care‐as‐usual, using a baseline measurement and follow‐up measurements after 7 and 14 months.

### 2.2 Study setting and participants

We performed a two‐stage sampling, first sampling intellectual disability‐care organizations and then assigning homes per organization to either the DCM or the control condition. First, we approached all six major intellectual disability‐care organizations which had at least four group homes for older clients in the north of the Netherlands; all were willing to participate (100%). Second, each organization provided four group homes for the study. A group home houses a small number (range 4 to 12) of older people with intellectual disability in need of care, support and supervision by care staff are living together. All participants were clients living in such group homes. The possibilities for using DCM determined our inclusion criteria for the group homes; we needed the possibility to observe four people simultaneously in a shared area (e.g., a living room) for at least two consecutive hours, the presence of at least three older people with (a strong suspicion of) dementia and a stable team not anticipating reorganization.

To reach a balance between groups regarding organizational culture, we allocated two of the four homes per organization to the intervention group and two to the control group. Allocation of group homes to the intervention or control groups depended on the distance between the mapper and the group home, and on sufficient geographic distance between control and intervention homes to prevent contamination.

### 2.3 Intervention

The intervention consisted of two applications of a full DCM cycle per group home, with an interval of six months. We used the DCM‐in‐ID–version, which was found to be feasible in intellectual disability care for older people with intellectual disability, both with and without dementia. In this version, the core DCM principles and DCM codes were maintained but the description of the codes was adapted to intellectual disability‐care practice (Schaap, Dijkstra et al., 2018; Schaap, Fokkens et al., 2018). First, the managers of each of the twelve participating group homes selected a staff member with the required competencies to become a “DCM mapper”, that is a trained observer. The twelve selected staff members had the required competencies, including at least 10‐year work experience with older people with intellectual disability, at least 5‐year work experience in working with people with dementia, at least a bachelor's degree, and basic knowledge of person‐centred care. DCM Netherlands trained these staff members to an advanced DCM level, enabling them to carry out DCM: to observe (map), report and provide feedback, and to instruct and support in drawing up action plans (Box 1) (Van de Ven et al., 2013). Second, a DCM trainer and a mapper jointly provided all staff per group home with the DCM introductory organizational briefing (see Box 1). Third, the mappers carried out two full DCM cycles, consisting of 6 hr structured observation, feedback and action planning (for further explanation see Box 1). The mappers observed four clients for 4 to 6 consecutive hours in communal areas of a group home. They reported the results of the observation to the staff in a feedback session, in order to help them understand clients’ behaviour in the context of their lives and of the care (Brooker & Surr, 2005). Based on these reports, the staff made action plans to improve care at individual and group levels. They sent these action plans to DCM Netherlands within two months. To guarantee accurate implementation, the application of DCM (including the feedback and the action plans) occurred in close cooperation with the DCM trainers. Further, to maintain independence and to avoid interpretation bias due to familiarity with habits, clients and colleagues of the mappers carried out DCM in each other's organizations.

To guarantee intervention adherence, the DCM trainers strictly monitored the intervention and supported the newly trained mappers in following the DCM‐in‐intellectual disability implementation protocol (Bradford Dementia Group, 2014). This protocol includes a description of all DCM preconditions and of every step needed to implement DCM in intellectual disability care (Bradford Dementia Group, 2014). This protocol ensured that DCM was implemented and applied similarly in each group home, in spite of differences in (staff‐team) size, number of residents, culture and approach.

### 2.4 Control condition

The control group received care‐as‐usual (CAU; continuous care with use of regular services); support in all aspects of day‐to‐day life, including activities of daily living (ADL) and day‐care activities) but no DCM. The control group homes were offered a DCM training day after the study period.

### 2.5 Procedure

We collected data on all clients living in the group homes, with or without dementia, at three time points: at baseline, and after 7 and 14 months (i.e., three months after each application of DCM in the intervention group). For each client in the group home, two staff members familiar with the client independently filled in a questionnaire at each time point. The inter‐observer agreement for each client at each time point was high (mean Kappa 0.81). In addition, for each client, we asked one relative to fill in the questionnaire. Staff and relatives could choose to fill in the questionnaire on paper or web‐based.

### 2.6 Outcome measures

The primary outcome measure regarded the quality of life (QoL) of the client as reported by staff and a close relative, measured by the Mood, Interest and Pleasure Questionnaire (MIPQ) (Petry, Kuppens, Vos, & Maes, 2010; Ross & Oliver, 2003). This validated questionnaire was chosen because it relates best to the core elements of DCM. The MIPQ measures emotional QoL of people with severe and profound intellectual and multiple disabilities, by using proxies. It is a 23‐item questionnaire using a five‐point Likert‐scale response format. All items regard informants’ observations of people over the preceding two‐week period. They are divided into three subscales: the “positive mood” subscale (9 items), the “negative mood” subscale (7 items) and the “interest & pleasure” subscale (7 items). Lower scores denote lower mood levels and lower levels of interest and pleasure. By summing the item scores, the maximum possible scores for the positive mood subscale, negative mood subscale, interest & pleasure subscale and total scale are 36, 28, 28 and 92, respectively. See Table 1 for further details of this questionnaire.

**Table 1:** Properties of used outcome measures

Name	Internal consistency	Inter‐rater reliability	Test–retest reliability	Mean (*SD*)	Validated in Dutch	Nr questions/nr answer possibilities (Likert)	Proxy version	Developed for:	Separate use of sub‐scales	Responsive to change	Previous use in DCM	Domains
Mood, Interest & Pleasure Questionnaire (MIPQ)^a^, ^b^	*α* ≥ 0.94	*r* ≥ 0.74	*r ≥ *0.90	62.03 (15.45)	✓	23/5	✓	People with severe/profound intellectual disability	✓	✓		Emotional quality of life with subscales: Mood (positive/negative) Interest & pleasure
Positive mood subscale	*α* ≥ 0.93	*r* ≥ 0.76	*r* ≥ 0.89	23.09 (7.29)		9/5						
Negative mood subscale	*α* ≥ 0.84	*r* ≥ 0.69	*r* ≥ 0.86	21.91 (4.64)		7/5						
Interest & pleasure subscale	*α* ≥ 0.89	*r* ≥ 0.69	*r* ≥ 0.90	17.03 (6.14)		7/5						
Questionnaire Quality of Living (VKvB)^c^, ^d^	N/A	N/A	N/A	N/A	N/A	37/4	✓	People with PIMD^e^	✓	✓		Used questions regarding: Behaviour of clients Self‐management of clients Knowledge of staff to individual clients Adaptations to care and/or environment
EuroQol 5 Dimensions (EQ5D)	*α ≥ *0.64	N/A	*r* ≥ 0.72	0.11 (0.39)	✓	5/5	✓	General population (validated for cognitive impairment)	✓	✓	✓	General health status
EuroQol Visual Analogue Scale (EQ‐VAS)	N/A	N/A	N/A	51.54 (21.47)	✓	1/100	✓	General population (validated for cognitive impairment)		✓	✓	Health status (0–100)

a Primary outcome.

b Petry et al. (2010).

c Secondary outcome.

d Retrieved from http://vkvb.cce.nl/vkvb/inschrijving.

Background characteristic.

Validated for cognitive impairment.

Diaz‐Redondo et al. (2014) and Wolfs et al. (2007).

e PIMD: Profound Intellectual and Multiple Disabilities.

The secondary outcome regarded adapted parts of the Quality of Living‐Questionnaire for people with Profound Intellectual and Multiple Disabilities (PIMD) at the Dutch Centre for Consultation and Expertise (CCE). This questionnaire was developed to gain insight into the care for people with Profound Intellectual and Multiple Disabilities (PIMD) (Centre for Consultation and Expertise (CCE), 2013). We used only those subscales of the Quality of Living‐Questionnaire that matched DCM's aims: the clients’ behaviour (10 items), self‐management (4 items), knowledge of staff about the individual client (15 items) and adaptations of staff and environment to respond to clients’ needs (8 items). All subscales used a four‐point Likert‐scale from “never” to “always” per item. The score on each subscale is the mean of the scores on all items, where higher scores denote better quality of living.

### 2.7 Background characteristics

Data on background characteristics of clients included age, sex, level of disability, dementia stages, having a syndrome, other (physical and mental) diseases and health status as measured by the EuroQol‐5D‐5L, including EQ‐5D‐VAS (Visual Analogue Scale) for proxies (Janssen et al., 2013). Furthermore, we registered the number of years that the clients were living in homes of the organization and in the group home concerned, whether the clients had day‐care activities in‐ or outside the group home, and whether the clients had contact with a relative.

In addition, we examined the background characteristics of the proxies (staff). These characteristics included age, gender, education, employment, job position, experience and training in person‐centred psychosocial approaches: Method Urlings, validation, reminiscence therapy, emotion‐oriented care and gentle care (Bakken, Sageng, Hellerud, Kildahl, & Kristiansen, 2017; Buijssen, 1991; Finnema, Dröes, Ribbe, & Van Tilburg, 2000; Van Puyenbroeck & Maes, 2008, 2009; Schrijnemaekers et al., 2002; Urlings, 2014).

### 2.8 Sample size

Because DCM is an intervention aimed at staff, the sample size for including group homes depended on the number of care staff required. We therefore conducted a post hoc power analysis for clients, using as outcome the Mood, Interest and Pleasure Questionnaire (MIPQ) (Petry et al., 2010; Ross & Oliver, 2003). A post hoc power analysis involves a power calculation based on the collected data to show specifically how much power the study has. This analysis of the difference in effects revealed low power (<0.8), particularly due to the small effect sizes found, which required large samples to detect. The post hoc power estimates were 0.11 and 0.07 for interaction term interventions by T1 and by T2, respectively. We performed power analysis using a Monte Carlo simulation of the MPlus package version 8.

### 2.9 Data analysis and reporting

First, we described the flow of clients. Second, we described the baseline characteristics of the clients in the two groups. We tested the differences between the two groups using Pearson chi‐square tests for categorical variables and one‐way analysis of variance (ANOVA) for continuous variables. Third, we compared the differences over time of the primary and secondary outcomes in the DCM and CAU groups. Because of the high inter‐observer agreement, we performed all analyses without further adjustments for informants. We assessed the effects of DCM using intention‐to‐treat (ITT) analyses after the first DCM cycle (T0 to T1) and after the second DCM cycle (T0 to T2). We did so using multilevel mixed‐effect model techniques in which measurement moments (level 3) were nested under clients (level 2), and the clients were nested under organizations (level 1). We performed the first analysis using the unconditional means model (Singer & Willett, 2003). For each outcome, we calculated effect sizes (ESs) for the differences in change between both groups. In this analysis, the time points were the first level, the clients the second and the group homes the third.

We repeated these analyses in three additional procedures. First, we included covariates found to have a significant influence on the intercept in the conditional means model, to examine whether this had a major influence on the outcomes. Covariates included age and sex, as well as prevalence of dementia, autism and/or of severe behavioural problems. Second, we performed complete case analyses only on those clients regarding whom we received questionnaires at all three time points. Third, we restricted the analyses to people with intellectual disability and a diagnosis of dementia. Finally, we examined whether the results differed depending on whether or not proxy informants had experience with a person‐centred approach.

We performed all analyses using IBM SPSS Statistics version 25.0; we used SAS software for data management. We carried out the design, analysis and reporting according to the CONSORT‐checklist (Schulz, Altman, & Moher, 2010).

### 2.10 Ethical permission

The Medical Ethical Committee of the University Medical Center Groningen did not consider approval to be required (decision M13.146536) because DCM is an intervention aimed at staff. We performed the trial in accordance with the Helsinki Declaration and obtained written informed consent from the legal representatives (i.e., a relative or an administrative person) of the people with intellectual disability participating in the study. The trial is registered in the Dutch Trial Register, number NTR2630.

## 3 RESULTS

### 3.1 Participant flow

**Figure 2:**
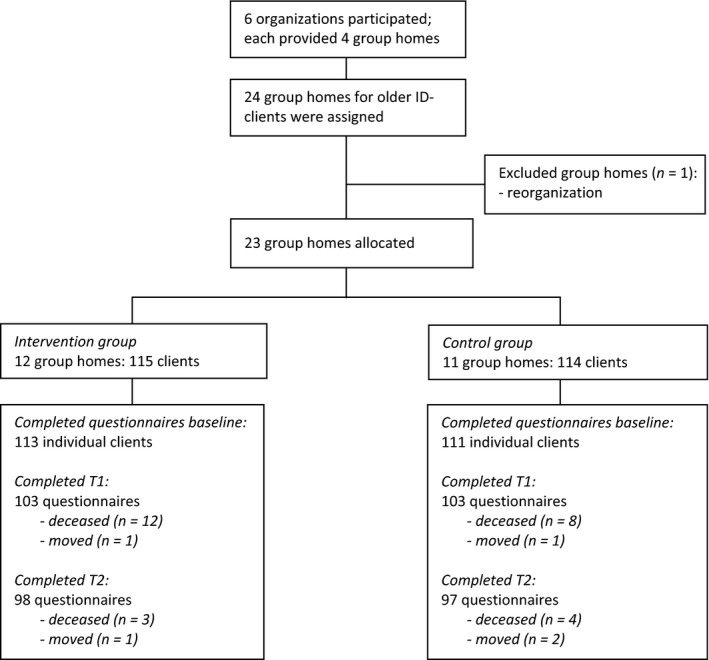
Flowchart detailing numbers of group homes and staff members by condition

Figure 2 shows the flow of clients through the study. In total, at least one baseline questionnaire was filled in for each of 224 clients, 113 in the intervention group and 111 in the control group. For each client, two staff members had filled in a questionnaire, but most relatives reported being unable to fill in the questionnaire because they did not see their relative on a daily basis. We therefore omitted the questionnaires of relatives from the analysis. After checking the inter‐observer agreement of staff for each client, we used all raw data for analysis. Inter‐observer agreement varied from 0.60 to 0.95, with a mean of 0.81; 0.41 of 0.60 indicates moderate agreement, 0.61 of 0.80 substantial agreement and 0.81 of 1.00 excellent, almost perfect agreement (Viera & Garrett, 2005).

### 3.2 Background characteristics

Clients in the intervention and control groups did not differ in background characteristics regarding age, gender, mean years in current location and having day‐care activities, but clients in the intervention group turned out to have more severe handicaps, more behavioural problems, more dementia and a lower health‐ and physical status (Table 2). Between the intervention and CAU groups, the background characteristics of the staff did not differ.

**Table 2:** Background characteristics of clients and of staff who reported on clients for the intervention (“DCM”) and care‐as‐usual (CAU) group

	DCM	CAU	*p*‐value
Clients			
*N*	113	111	
Mean age in years (*SD*)	67 (11.3)	65 (12.4)	0.38
Female (%)	43	56	0.05
Mean years in current organization (*SD)*	31 (15.6)	27 (13.8)	0.05
Mean years in current location (*SD*)	8 (5.9)	10 (8.2)	0.033
Handicap (%)			
Mild	21	31	0.004*
Moderate	49	56	
Severe/Profound	31	13	
Dementia (%)			
Diagnosed	35	17	0.004*
Suspicion	11	7	
Signs of	18	22	
Autism	28	29	0.85
Psychiatric disease	22	17	0.40
Challenging behaviour (%)	31	29	0.69
Severe behavioural problems (%)	5	13	0.034*
Mobility/motor problems (%)	53	41	0.07
Communication problems (incl. sight and hearing) (%)	66	45	0.002*
Health problems (incl diabetes) (%)	58	44	0.037*
Mean EQ5D – total (*SD*)	2.68 (0.78)	2.34 (0.70)	0.001*
Mean EQ5D VAS (*SD*)	66.6 (10.8)	66.4 (13.0)	0.31
Day‐care activities (%)	95	95	0.93
Unknown life‐history (%)	19	14	0.30
Need for knowledge about client (%)	47	38	0.17
Staff			
*N*	85	75	
Mean age in years (*SD*)	48 (11.7)	47 (11.9)	0.78
Female (%)	90	90	0.50
Education			
Only elementary and secondary education (%)	9	9	0.75
Secondary vocational education (%)	80	77	
Higher professional education (%)	11	13	
Position			
Daily care professional (%)	71	75	0.40
Senior‐/coordinating care professional/personal coach (%)	24	23	
Permanent employment (%)	90	93	0.82
Hours/week (mean)	23	24	0.86
Experience			
>11 years in intellectual disability care (%)	71	69	0.63
>11 years in current group home (%)	35	31	
Education of older intellectual disability‐clients (%)	76	69	0.29
Training in person centred psychosocial approach/method^a^ (%)	35	35	0.92

a These regarded: Method Urlings (Urlings, 2014), Validation (Bakken et al., 2017), reminiscence therapy (Van Puyenbroeck & Maes, 2008, 2009), emotion‐oriented care (Finnema et al., 2000; Schrijnemaekers et al., 2002) and gentle Care (Buijssen, 1991).

* significant difference between DCM and CAU (P=<0.05)

### 3.3 Effects on primary and secondary outcomes

Table 3 presents the effects of DCM compared to CAU. We found no differences in change for of the primary outcome (MIPQ) between T0 and T1, and between T0 and T2. Effect sizes varied from 0.01 to 0.05 for T0 to T1, and from 0.01 to −0.15 for T0 to T2. Regarding secondary outcomes, we also found no differences between T0 and T1, and between T0 and T2. Effect sizes varied from 0.01 to 0.10 for T0 to T1, and from −0.09 to −0.22 for T0 to T2.

**Table 3:** Outcomes for DCM and CAU at T0, T1 and T2: means and differences in improvement, based on intention‐to‐treat analyses with mixed multilevel models (*n* = 224)

Outcome	Group	T0 (Baseline)		T1 (Three months after 1st DCM cycle		Difference in improvement T0 to T1 between DCM and CAU			T2 (Three months after 2nd DCM Cycle		Difference in improvement T0 to T2 between DCM and CAU		
		Mean	*SD*	Mean	*SD*	Dif^d^	*p*‐value	ES^e^	Mean	*SD*	Dif^d^	*p*‐value	ES^e^
MIPQ^a^, ^b^	DCM	83.77	17.66	83.11	17.09	0.57	0.69	0.03	82.77	16.79	0.15	0.91	0.01
	CAU	85.08	17.50	83.85	17.06				83.93	16.65			
Positive mood	DCM	30.92	8.32	30.73	8.08	0.37	0.61	0.05	30.46	7.86	−0.26	0.72	−0.03
	CAU	31.06	8.24	30.50	8.05				30.86	7.78			
Negative mood	DCM	23.90	7.44	23.42	7.19	0.08	0.88	0.01	23.92	6.90	0.95	0.11	0.13
	CAU	24.82	7.42	24.26	7.22				23.89	6.89			
Interest/pleasure	DCM	28.94	3.78	28.96	3.72	0.11	0.80	0.03	28.41	3.57	−0.55	0.23	−0.15
	CAU	29.19	3.72	29.10	3.67				29.21	3.50			
Behaviour of client^c^	DCM	3.19	0.51	3.14	0.50	−0.03	0.40	−0.07	3.12	0.46	−0.09	0.07	−0.19
	CAU	3.13	0.51	3.11	0.50				3.15	0.46			
Client's self‐management^c^	DCM	2.92	0.79	2.93	0.76	−0.04	0.62	−0.05	2.92	0.71	−0.07	0.38	−0.09
	CAU	3.04	0.79	3.09	0.77				3.11	0.72			
Knowledge about client^c^	DCM	3.08	0.82	3.14	0.79	0.08	0.20	0.10	3.08	0.73	−0.11	0.09	−0.14
	CAU	3.14	0.83	3.13	0.81				3.26	0.75			
Adaptations to the client^c^	DCM	3.11	0.69	3.09	0.66	0.01	0.91	0.01	3.00	0.60	−0.14	0.06	−0.22
	CAU	3.17	0.70	3.14	0.68				3.21	0.61			

a Primary outcome.

b Mood, Interest and Pleasure Questionnaire.

c Secondary outcome.

d Based on mixed model techniques, expressing differences in change between DCM and CAU in outcomes.

e Effect size (Cohen's *d*).

Adjustment for the covariates did not lead to notable changes in the results, nor did complete case analysis. Repeating the analysis including only people with a diagnosis of dementia led to slightly lower means on all outcomes for each time point (decrease varying from 2.87 to 5.72 on the total score of MIPQ and 0.06 to 0.26 on the secondary measures), but did not significantly affect differences in outcomes. Findings did not differ between staff experienced with person‐centred care and staff without this experience.

## 4 DISCUSSION

This study examined the effectiveness of the intervention DCM on quality of life and well‐being of older people with intellectual disability. We found no significant differences in effects between DCM and CAU on the outcomes; effect sizes were small (Cohen, 1988).

In this well‐designed quasi‐experimental study, we found a lack of effect of DCM on quality of life, a result which contrasts with promising findings in earlier qualitative studies on DCM and person‐centred intellectual disability care (Finnamore & Lord, 2007; Jaycock et al., 2006; Schaap, Dijkstra et al., 2018; Schaap, Fokkens et al., 2018). This may be explained in several ways. First, we found rather high scores on most outcome measures at baseline, which may have caused a ceiling effect in measuring effects. For example, on the primary outcome, MIPQ clients scored more than one standard deviation higher than the norm population (Petry et al., 2010); the same held to a slightly lesser degree for clients with a diagnosis of dementia. Staff members, the informants regarding client outcomes, may in general have been too positive.

Second, DCM requires a strong existing embedding of person‐centred care. Because this emphasis has evolved only recently in the field of intellectual disability care (Cleary & Doody, 2017; Van der Meer et al., 2018; Ratti et al., 2016), a comprehensive shared knowledge base among staff about person‐centred care and dementia is lacking. This indicates room for improvement by full implementation of person‐centred care in intellectual disability care for clients at different levels, as well in staff‐training (staff level), culture and organization of care (group home level) and the organizations’ underlying visions (management and organizational level) (Bertelli et al., 2017; Dowling, Manthorpe, & Cowley, 2007).

Third, DCM may simply not lead to a better quality of life. As in previous studies on DCM in intellectual disability care (Barbosa, Lord, Blighe, & Mountain, 2017; Dichter et al., 2015; Kuiper et al., 2009; Rokstad et al., 2013; Van de Ven et al., 2013), we have based our choice of outcome measures on DCM's claim that it increases the quality of life of clients as a result of improved quality of care. However, DCM may be a too light and too indirect intervention to directly affect quality of life, even if improving quality of care. In previous studies, staff claimed that they benefitted from DCM in daily care, although compliance to the action plans could be improved, as well as the provision of time and resources by management to staff (Schaap, Dijkstra et al., 2018; Schaap, Fokkens et al., 2018). This discrepancy deserves further study: what effect does DCM have on quality of care, and what effect does this then have for staff and clients in daily care? In addition, quality of life is a broader concept than might be influenced by DCM (pain, decline through ageing). Given the strength of our study, the lack of effects on staff‐reported quality of life of clients definitely requires further attention.

### 4.1 Strengths and limitations

Our study has a number of strengths. First, we carefully assessed the feasibility of DCM for intellectual disability care prior to this study with a positive result and used this adapted DCM‐in‐ID version (Schaap, Fokkens et al., 2018). Next, our study had a large sample size, a control group receiving CAU, participants from a wide range of organizations, sufficient strategies to avoid contamination and bias, and a long follow‐up of one year with two follow‐up measurements. Furthermore, our study had a low loss to follow‐up. Finally, the inter‐observer agreement between the proxies (two staff members) for the individual clients was high and perceived as good to excellent (Essen 2004; Viera & Garrett, 2005).

Limitations should, however, also be noted. First, we fully relied on reports of the staff, using proxy‐questionnaires; this may have led to information bias and a less accurate measurement of change. Moreover, relatives generally reported being unable to assess clients’ outcomes because they had no contact on a daily basis. Furthermore, due to chance we had some imbalances between the intervention and control groups, with relatively more severe disabilities and more dementia in the intervention group. However, given the pre‐post design that we used, this is unlikely to have affected our findings.

### 4.2 Implications

We found no evidence that DCM improves the quality of life of older people with intellectual disability. As previous qualitative studies are definitely positive regarding DCM (Schaap, Dijkstra et al., 2018; Schaap, Fokkens et al., 2018), further research is needed to elucidate this discrepancy, for example by means of in‐depth interviews with participating intellectual disability staff or direct observation. Furthermore, it is uncertain whether DCM affects quality of life directly, despite its own claim. Future research should investigate the effects of DCM in daily care and its direct effects on intellectual disability‐care staff and their clients. Moreover, the challenges of developing person‐centred care in intellectual disability care, including in the integration of health and social care, require better understanding (Bertelli et al., 2017). The promising option of DCM in intellectual disability care thus deserves further study.

## 5 CONCLUSION

Despite previous studies that reported that DCM and person‐centred care increases well‐being of older people with intellectual disability, with or without dementia, we have found no evidence that this is the case regarding quality of life. This discrepancy deserves further study.

## CONFLICT OF INTEREST

The authors have no conflict of interest to declare.
